# Effect of a self-developed fixation device on preventing endotracheal intubation-related pressure injury: a randomised controlled trial

**DOI:** 10.1186/s13054-024-04874-7

**Published:** 2024-03-19

**Authors:** Xiaodong Zhang, Qibing Zhang, Jiqin You, Rong Xu, Zhaojie Zhang, Yunlan Shi, Chunrong Han, Shiyan Zhao, Bangtao Yao, Yan Geng, Songqiao Liu

**Affiliations:** 1 Nanjing Lishui People’s Hospital, Zhongda Hospital Lishui Branch, Southeast University, Nanjing, 211200 China; 2 The Central Hospital of Xuzhou, Xuzhou, 221000 China; 3 Jiangsu Provincial Key Laboratory of Critical Care Medicine, Zhongda Hospital, School of Medicine, Southeast University, Nanjing, 210009 China

**Keywords:** Endotracheal tube, Fixation, Pressure injury, EIRPI, Lip

## Abstract

**Objective:**

To evaluate the effects of our self-developed endotracheal tube fixation device in mechanically ventilated patients.

**Methods:**

In a dual-centre randomised controlled trial, patients who were expected to require mechanical ventilation for over 48 h were assigned to the observation group (using self-developed device) or the control group (using the traditional device). The primary endpoint was the incidence of endotracheal intubation-related pressure injury (EIRPI).

**Results:**

Fifty-one patients in the observation group and 54 patients in the control group were analysed. The incidence of EIRPI was 7.8% in the observation group and 33.3% in the control group (*p* = 0.001). Lip pressure injury (PI) occurred in 0 versus 14 (25.9%) patients in the observation versus control groups (*p* < 0.001). Both oral–mucosal and facial PIs were similar between the two groups.

**Conclusions:**

The use of the novel device reduced the incidence of EIRPI, especially lip PI.

*Trial registration* Chinese Clinical Trial Registry ChiCTR2300078132. Registered on 29 November 2023

## Introduction

Endotracheal intubation through the oral cavity is the main method used to establish an artificial airway in critically ill patients [1, 2]. Effective fixation of the endotracheal tube is crucial to ensure respiratory function. Common clinical methods for fixing endotracheal tubes include tape, ropes, and commercially available fixation devices, which often lead to complications such as lip pressure injury (PI) [3] and unplanned extubation [4]. These complications cause suffering, prolong hospital stays, and increase healthcare expenses [5–7]. Therefore, improving the design of fixation devices is of considerable importance.

Thus, our team developed a novel device, which was authorised by the China National Intellectual Property Administration (patent no.: ZL202221745523.5). In this prospective study, we aimed to evaluate the effects of the novel device in mechanically ventilated patients. To the best of our knowledge, randomised controlled trials comparing PIs associated with endotracheal tube fixation devices from a design perspective are rare.

## Methods

### Study design

**Fig. 1:**
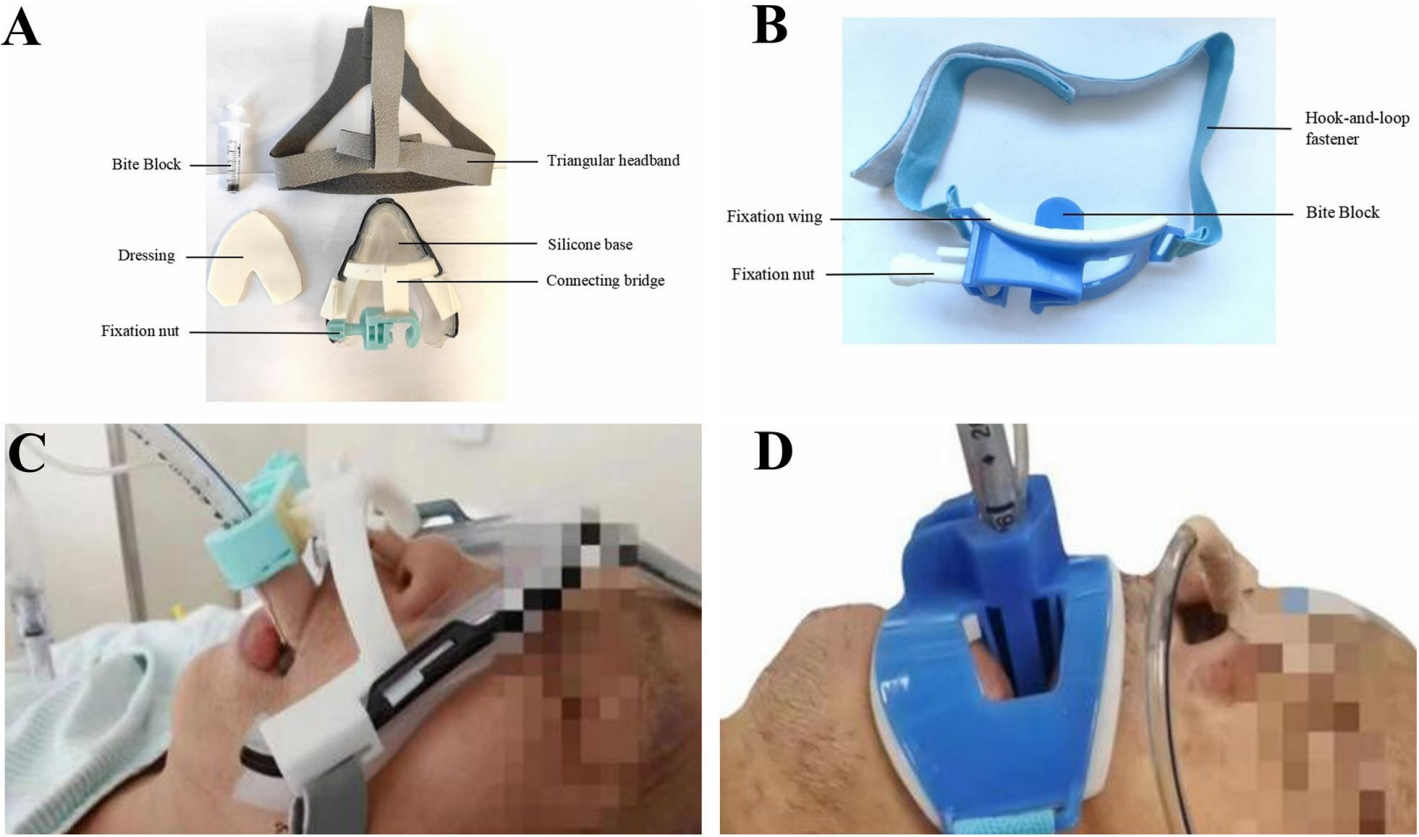
**A** Structure diagram of the self-developed endotracheal tube fixation device. **B** Structure diagram of the traditional endotracheal tube fixation device. **C** Photograph of the patient wearing the self-developed endotracheal tube fixation device. **D** Photograph of the patient wearing the traditional endotracheal tube fixation device

This prospective dual-centre randomised controlled trial aimed to compare the effect of a novel device (Fig. 1A) versus a traditional device (Zhejiang Haisheng Medical Equipment Co., Ltd.) (Fig. 1B) among critically ill adults requiring intubation and mechanical ventilation for at least 48 h. This study was approved by the Institutional Review Board of Nanjing Lishui People’s Hospital and the Medical Ethics Committee of Xuzhou City Central Hospital.

### Study participants

Patients admitted to the intensive care unit (ICU) of the two hospitals from 14 November 2022 to 20 October 2023 who required endotracheal intubation and met the study’s inclusion criteria were randomly allocated to two groups: the observation group with the novel device (Fig. 1C) and the control group with the traditional device (Fig. 1D).

The inclusion criteria were as follows: (1) age ≥ 18 years; (2) meeting the indications for endotracheal intubation; and (3) signing the informed consent by themselves or their family member.

The exclusion criteria were as follows: (1) patients who were not suitable for orotracheal intubation; (2) patients with damaged lip, facial skin, or oral mucosa before intubation; and (3) patients who were not suitable for wearing headbands.

Withdrawal criteria were as follows: (1) patients with intubation time/mechanical ventilation time < 48 h, (2) patients who abandoned the treatment during the trial, and (3) patients or family members who voluntarily requested withdrawal during the trial.

### Randomisation

Patients were randomly allocated to the observation or control group by a person other than the researchers in a 1:1 ratio with random sequences generated using IBM SPSS Statistics for Windows, version 25.0 (IBM Corp., Armonk, NY, USA), stratified by centre. Allocation information was concealed using sealed opaque envelopes.

### Study outcomes

The primary outcome was the appearance of any endotracheal intubation-related pressure injury (EIRPI) that occurred on the lips, oral mucosa, or facial skin during intubation. Secondary outcomes included duration of mechanical ventilation, tube dislodgement, and ventilator-associated pneumonia (VAP).

### Statistical analyses

We estimated the expected incidence of EIRPI to be 28% in the control group and 5% in the observation group based on the data from our preliminary test. The sample size was calculated using PASS software, version 15.0.5 (NCSS, LLC., Kaysville, Utah, USA) with 90% power and a two-sided alpha level of 0.05. Considering a 10% dropout rate, we planned to randomise 112 patients to achieve the 100 eligible patients required for our study.

All hypothesis tests were two-sided, and the threshold for significance was set to 0.05. The Shapiro–Wilk test was used to assess the normality of the distribution. Non-normally distributed continuous variables were presented as medians (quartile 1 and quartile 3), and the Mann–Whitney U-test was used to assess differences between the two groups. The proportions of categorical variables were compared using the Chi-squared test or Fisher’s exact test, as appropriate. All analyses were performed using IBM SPSS Statistics for Windows, version 25.0.

## Results

### Baseline characteristics

**Fig. 2:**
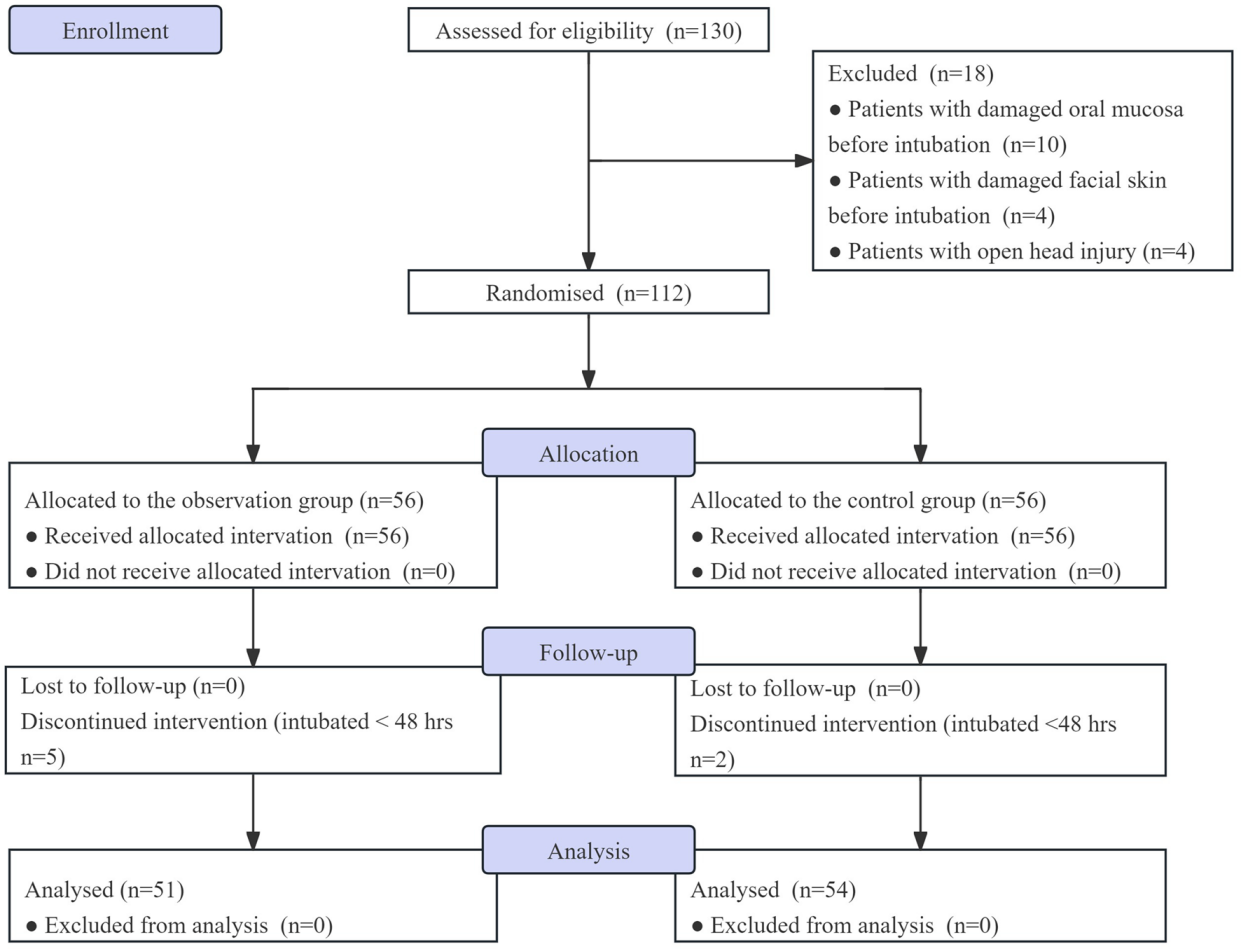
Flow diagram of enrolment and randomisation of patients

Inclusion, exclusion, and withdrawal criteria resulted in 51 patients in the observation group and 54 patients in the control group (Fig. 2). Baseline characteristics between the two groups showed no significant differences (Table 1).

**Table 1:** Characteristics of patients at baseline

Characteristic	Observation group (*n* = 51)	Control group (*n* = 54)	*p* value
Age (years)	68.0 (56.0, 78.0)	70.0 (60.0, 79.0)	0.445
Male sex—no. (%)	34 (66.7)	36 (66.7)	> 0.999
Height (cm)	170.0 (160.0, 175.0)	170.0 (161.8, 175.0)	0.591
Weight (kg)	68.0 (60.0, 75.0)	69.0 (60.0, 73.5)	0.648
BMI	23.7 (22.0, 25.5)	24.2 (21.4, 25.9)	0.977
APACHE II	21.0 (18.0, 23.0)	20.0 (17.0, 23.3)	0.985
Indication for intubation—no. (%)			
Respiratory failure	19 (37.3)	28 (51.9)	0.475
Altered mental status	13 (25.5)	10 (18.5)	
Airway obstruction	2 (3.9)	2 (3.7)	
Haemodynamic instability	17 (33.3)	14 (25.9)	

### Primary outcome

The primary endpoint occurred five times in four (7.8%) patients in the observation group and 29 times in 18 (33.3%) patients in the control group (*p* = 0.001), with an overall incidence rate of 16.8 versus 88.1 per 1000 ventilator days, in the observation versus control groups, respectively (*p* < 0.001). Lip PI occurred in 0 versus 14 (25.9%) patients, with an incidence rate of 0 versus 63.8 per 1000 ventilator days in the observation and control groups, respectively (*p* < 0.001). Both oral–mucosal and facial PIs showed no significant differences between the two groups (Table 2).

**Table 2:** Clinical outcomes

Outcome	Observation group (*n* = 51)	Control group (*n* = 54)	*p* value
Primary outcome Pressure injury on the lips, oral mucosa, or facial skin—no. of patients (%) Rate of primary outcome (per 1000 patient ventilator days) (95% confidence interval)	4 (7.8) 16.8 (2.2, 31.5)	18 (33.3) 88.1 (57.5, 118.8)	0.001 < 0.001
Lip pressure injury—no. (%) Rate per 1000 patient ventilator days	0 0	14 (25.9) 63.8 (37.4, 90.2)	< 0.001 < 0.001
Oral–mucosal pressure injury—no. (%) Rate per 1000 patient ventilator days	2 (3.9) 6.7 (−2.6, 16.0)	5 (9.3) 15.2 (2.0, 28.4)	0.481 0.532
Facial pressure injury—no. (%) Rate per 1000 patient ventilator days	3 (5.9) 10.1 (−1.3, 21.4)	3 (5.6) 9.1 (−1.2, 19.4)	> 0.999 > 0.999
Secondary outcomes Tube dislodgement—no. (%)	0	0	N/A
Ventilator-associated pneumonia—no. (%)	0	2 (3.7)	0.496
Duration of mechanical ventilation (days)	5.0 (3.0, 8.0)	5.0 (3.8, 8.3)	> 0.999

### Secondary outcomes

No differences were observed between the two groups in terms of the duration of mechanical ventilation, VAP, or tube dislodgement (Table 2).

## Discussion

In this study, the observation group had a significantly lower incidence of EIRPI compared to the control group. The incidence of lip PI was significantly reduced, but there were no significant differences in oral–mucosal or facial PIs.

Recent studies have reported different rates of PIs with traditional endotracheal tube fixation devices. Sun et al. reported that the incidence of facial and lip PIs was 58.1% [8]. Qin et al. reported that the incidence of EIRPI was 23.7% and that the lip was the most commonly affected area (76.7%) [9]. In our study, the incidence of EIRPI with a traditional device was 33.3%, which is comparable to the findings of the aforementioned two studies. We hypothesised that the variation in reported incidence may be due to differences in headband tension, as there were no devices to measure and tailor it for each patient.

The National Pressure Ulcer Advisory Panel recognises the vulnerability of mucosal tissue to PIs caused by medical devices such as endotracheal tube fixation devices, emphasising the potential for tissue ulcers due to applied pressure [10]. In this study, the incidence of EIRPI was 8.7% in the observation group and 33.3% in the control group. The results confirmed that the novel device had significant advantages in preventing EIRPI, particularly lip PI.

We conclude the reasons for this to be:

- Suitability of the contact area: The contact of the novel device with the skin of the face compared to the lips, it is less prone to mechanical damage. The facial skin has a higher degree of keratinisation, which provides stronger protective function [11].
- Differences in the environment at the contact areas: The complex environment of the lips, including factors such as oral secretions and bacteria, increases the risk of PI. The moist environment of the lips weakens the mucosal barrier function [12] and predisposes to secondary infection. On the other hand, the environment of the facial skin is simpler, and dressings placed at the skin–silicone interfaces can prevent the accumulation of skin moisture, reducing the occurrence of PI [13].
- The use of silicone pads and dressings in the novel device promotes reduced and uniform pressure between the skin and device, thereby reducing the occurrence of PI [14–16].

Overall, the advantages of the novel device include shifting the point of force application away from the lips prone to EIRPI, minimising the risk of mechanical damage to the lips.

In our previous study, the novel device was used to successfully treat lip PI in a 79-year-old patient with diabetes [17].

This study has several limitations, including a dual-centre design, small sample size, limited research duration, and imprecise control of headband tension, and the control group is not a standard of care in every ICU. Moreover, the open-label study design with a subjective outcome, i.e. the number of PIs, could introduce bias into the findings. Future research should aim to improve methodology by integrating more objective assessment tools.

To improve understanding of the novel device used in mechanically ventilated patients, multi-centre studies are needed. Future research should investigate incorporating sensors into endotracheal tube fixation devices to monitor real-time data on skin conditions, blood perfusion, and tissue cell deformation, potentially reducing the incidence of EIRPI.

## Conclusions

This study demonstrated that the novel device has therapeutic potential in preventing EIRPI, especially lip PI.

## Data Availability

The datasets generated and/or analysed during the current study are available from the corresponding author on reasonable request.

## Abbreviations

EIRPI: Endotracheal intubation-related pressure injury
PI: Pressure injury
ICU: Intensive care unit
VAP: Ventilator-associated pneumonia
